# Lipopolysaccharide differentially affects the osteogenic differentiation of periodontal ligament stem cells and bone marrow mesenchymal stem cells through Toll-like receptor 4 mediated nuclear factor κB pathway

**DOI:** 10.1186/scrt456

**Published:** 2014-05-27

**Authors:** Chenghua Li, Bei Li, Zhiwei Dong, Li Gao, Xiaoning He, Li Liao, Chenghu Hu, Qintao Wang, Yan Jin

**Affiliations:** 1 State Key Laboratory of Military Stomatology, Department of Periodontology, School of Stomatology, The Fourth Military Medical University, Xi’an, Shaanxi 710032, China; 2 State Key Laboratory of Military Stomatology, Center for Tissue Engineering, School of Stomatology, The Fourth Military Medical University, Xi’an, Shaanxi 710032, China; 3 Research and Development Center for Tissue Engineering, Fourth Military Medical University, Xi’an, Shaanxi 710032, China; 4 Department of Oral and Maxillofacial Surgery, General Hospital of Shenyang Military Area Command, Shenyang, Liaoning 110840, China

## Abstract

**Introduction:**

Periodontitis is initiated and sustained by bacteria. However, the mechanism of bacteria induced periodontitis is still unknown. We hypothesized that bacterial components can affect the functions of stem cells in the periodontium. In this study, we comparatively investigated the influence of Lipopolysaccharide (LPS) on the osteogenesis potential of human periodontal ligament stem cells (PDLSCs) and bone marrow mesenchymal stem cells (BMMSCs).

**Methods:**

Human PDLSCs and BMMSCs were harvested and mineralized nodule formation was assessed by alizarin red S staining. Expression level of osteogenic related gene was detected by quantitative RT-PCR (qRT-PCR). The expression of Toll-like receptor 4 (TLR4) and its downstream signaling pathway were examined by western blot. The role of TLR4 and related signaling pathway in LPS impairing the osteogenic potential of human PDLSCs and BMMSCs were also studied by alizarin red S staining and qRT-PCR. Experimental periodontitis was induced in adult Sprague–Dawley rats and the alveolar bone loss was measured by micro computed tomography analysis. The expression of alkaline phosphatase (ALP) was assessed by immunohistochemistry and the number of osteoclasts was shown by Tartrate-resistant acid phosphatase (TRAP) staining.

**Results:**

LPS decreased the osteogenic differentiation of human PDLSCs through TLR4 regulated nuclear factor (NF)-κB pathway, but not for BMMSCs. Blocking TLR4 or NF-κB signaling partially reversed the decreased osteogenic potential of PDLSCs and prevented the alveolar bone loss caused by LPS experimental periodontitis in rats. The ALP expression in the periodontal ligament was elevated after treatment with anti-TLR4 antibody or pyrrolidinedithiocarbamate, whereas there was no statistical significance among groups for the number of osteoclasts.

**Conclusions:**

These data suggest that LPS can activate TLR4 regulated NF-κB pathway of human PDLSCs, thus decreasing their osteogenic potential. Blockage of TLR4 or NF-κB pathway might provide a new approach for periodontitis treatment.

## Introduction

Periodontitis is characterized by the inflammatory reaction of the surroundings of the teeth, mostly caused by an oral microbial biofilm and perpetuated by an uncoordinated immune-inflammatory response, which ultimately leads to progressive destruction of the tissues supporting the teeth [1]. It is until now the major cause of tooth loss and is associated with a number of systemic diseases, such as diabetes and cardiovascular diseases [2], while no appropriate method has been developed to provide a functional and predictable method for periodontal regeneration. Lipopolysaccharide (LPS), a cell wall component of gram-negative bacteria, is mainly recognized by toll-like receptor 4 (TLR4) of the host. This bimolecular compound penetrates periodontal tissue [3,4] and is considered to be a major nexus for virulence in periodontitis [5,6]. Previously, numerous studies have been conducted to examine the role of LPS in periodontal pathogenesis. However, the underlying molecular mechanism of LPS-host interaction is still unclear.

Mesenchymal stem cells play a key role in the maintenance of the regenerative capacity of periodontal tissue. The discovery of periodontal ligament stem cells (PDLSCs), which form a cementum/PDL-like structure after *in vivo* transplantation, provides a new prospect for periodontal tissue regeneration [7]. After transplantation, PDLSCs effectively regenerated the alveolar bone in the defects created by surgical bur in miniature pigs, showing encouraging results in preclinical trials [8,9]. In addition, bone marrow mesenchymal stem cells (BMMSCs), originated from bone marrow, also have been documented to possess the capacity to regenerate periodontal tissue in various animal models [10,11]. However, in a diseased periodontal environment, tissue repair does not occur naturally because of the lack of robust stem cells, which leads to the loss of periodontal tissue including cementum/periodontal ligament and the alveolar bone [12]. Repair of the alveolar bone is considered to be controlled by the stem cells in the niche area, such as PDLSCs or BMMSCs. However, the toxic product of bacteria, LPS, is elevated in the oral cavity of periodontitis patients [13] and it may affect the bone regeneration capacity of PDLSCs and BMMSCs.

Up to now, controversial findings have been reported regarding the role of LPS and TLR4 in the osteogenic differentiation of BMMSCs [14-16]. In addition, there are still no reports on the expression of TLR4 in PDLSCs and the influence of LPS on the osteogenic differentiation of PDLSCs. In this study, we sought to comparatively investigate the influence of LPS on the osteogenesis potential of PDLSCs and BMMSCs and further explore the mechanisms of LPS regulation of the osteogenic differentiation of these two kinds of MSCs. The results indicated that LPS decreased the osteogenic potential of PDLSCs through the TLR4 regulated NF-κB pathway, but not that of BMMSCs. Blocking the TLR4 or NF-κB pathway partially reversed the impaired osteogenic potential of PDLSCs after LPS treatment and prevented the alveolar bone loss induced by LPS in experimental periodontitis in rats.

## Materials and Methods

### Isolation of PDLSCs

Healthy human third molars were extracted from five systemically healthy adults (25 to 30 years of age) at the Department of Periodontology and Oral Medicine, Stomatological Hospital of the Fourth Military Medical University. Written consent was obtained from them prior to conducting the study. Ethical approval had been obtained from the Ethics Committee of the School of Stomatology, Fourth Military Medical University.

PDLSCs were isolated and cultured as we previously described [17,18]. PDL (periodontal ligament) tissues were scraped off the middle third of the root surface and then digested with collagenase I (3 mg/ml; Sigma–Aldrich, St. Louis, MO, USA) for two hours at 37°C to obtain single cell suspensions. Cells were maintained in α-minimal essential medium (α-MEM; Sigma–Aldrich) with 10% fetal bovine serum (FBS; Thermo Electron, Melbourne, Australia), 2 mM L-glutamine, 100 U/ml penicillin and 100 mg/ml streptomycin, and incubated at 37°C in 5% CO_2_. The medium was changed every three days. Single cell-derived colony cultures were obtained using the limiting dilution technique, and different colonies were gathered as passage 0 (P0) cells. To avoid changes in cell behavior caused by prolonged culture, only cells from P3 to P5 were used in this study.

### Isolation of BMMSCs

Bone marrow was harvested from the iliac crest of three healthy volunteers (25 to 30 years of age). Written consent was obtained from them prior to conducting the study. Ethical approval had been obtained from the Ethics Committee of the School of Stomatology, Fourth Military Medical University. Mononuclear cells were isolated by density gradient centrifugation (TBD, Tianjin, China), washed in PBS and seeded at 2 × 10^4^ cells/cm^2^ in α-MEM (Sigma–Aldrich) with 10% FBS (Thermo Electron), 2 mM L-glutamine, 100 U/ml penicillin and 100 mg/ml streptomycin, and incubated at 37°C in 5% CO_2_. After 48 hours, non-adherent cells were removed by washing and the medium was changed every three days. At sub-confluence, the cells were detached by 0.25% trypsin and counted. For passages, cells were replated in 75 cm^2^ flasks in the same culture conditions until sub-confluence. The cells of passage 3 to 5 were used for the following experiments.

### Total RNA extraction and quantitative RT-PCR

Total cellular RNA was extracted by TRIzol reagent (Invitrogen, Eugene, OR, USA) according to the manufacturer’s instructions. Isolated total RNA was then subjected to reverse transcription using OligodT primer and PrimeScript® RTase (Takara, Dalian, China) according to the manufacturer’s instructions. Quantitative RT-PCR (qRT-PCR) was performed with SYBR® Premix Ex Taq™ II (Takara, Dalian, China) using the C1000TM Thermal Cycler (Bio-Rad, Hercules, CA, USA).

The primers used in this study were as follows: TLR1: forward, 5′-**CAGTTACTCCCGGAGGCAATGCT**-3′, and reverse, 5′-**AGATTCCTTTTGTAGGGG TGCCCA**-3′; TLR2: forward, 5′-**TTGTGCCCATTGCTCTTTCA**- 3′, and reverse, 5′-**GCTTCAACCCACAACTACCAGTT**-3′; TLR3: forward, 5′-**CACGGCTCTGGAAACA CGCA**-3′, and reverse, 5′-**AGGTTCCTGAAAGCTGGCCCGA**-3′; TLR4: forward, 5′- **ACCTGATGCTTCTTGCTGGCTGC**- 3′, and reverse, 5′-**AGCAATGGCCACACCGG GAA**-3′; TLR5: forward, 5′-**CCTCTGCCCCTAGAATAAGAACATA**-3′, and reverse, 5′-**TGATCCTCGTTGTCCTAGCAGAA**-3′; TLR6: forward, 5′-**AGCCACTGCAACATCA TGACCAA**- 3′, and reverse, 5′-**TGTCAGAGACCTGAAGCTCAGCGA**-3′; TLR7: forward, 5′-**GGAAATTGCCCTCGTTGTTA**-3′, and reverse, 5′-**CTGGGGAGAAAATG CAGAAA**-3′; TLR8:forward, 5′-**GAGTTATGCGCCGAAGAAAATT**-3′, and reverse, 5′-**TTTCTCATCACAAGGATAGCTTCTAGAA**-3′; TLR9: forward, 5′-**AGGCCTGAGGC GGTTTGATCT**-3′, and reverse, 5′-**GGTGTGCAGGCGGTTCTG**-3′; TLR10: forward, 5′-**GGCCAGAAACTGTGGTCAAT**- 3′, and reverse, 5′-**AACTTCCTGGCAGCTCTGA A**-3′; Runx2: forward, 5′-**CCCGTGGCCTTCAAGGT** -3′, and reverse, 5′- **ATGACAGT ACCGCCCATTGC**- 3′; β-actin: forward, 5′-**CTCCACCCTGGCCTCGCTGT**-3′, and reverse, 5′- **GCTGTCACCTTCACCGTTCC**- 3′. The expression levels of the target genes were normalized to that of the housekeeping gene β-actin.

### *In vitro* osteogenic assay

PDLSCs or BMMSCs were analyzed for their capacity to differentiate toward osteogenic lineages. Cells were seeded in six-well culture plates at a density of 2 × 10^5^ cells/well. Osteogenic medium was α-MEM supplemented with 10% FBS, 100 nM dexamethasone, 5 mM β-glycerophosphate (Sigma-Aldrich) and 50 μg/ml L-ascorbic acid (Sigma-Aldrich). Cells cultured in induction medium additionally supplemented with LPS (10 μg/ml, O55:B5, Sigma-Aldrich) were used as the experimental group. The induction medium was changed every three days. At day 14, total RNA was extracted for the analysis of osteogenic gene (*Runx2*) by qRT-PCR. At day 28, the samples were fixed with 4% polyoxymethylene for 20 minutes. The osteogenic differentiation was assessed using Alizarin red S (Sigma-Aldrich) staining. For Alizarin red quantification, 1 ml of 10% cetylpyridinium chloride was added to each well. Light absorbance of the extracted dye was measured at 620 nm.

### Protein isolation and western blot analysis

Total proteins were extracted with lysis buffer (10 mM Tris–HCl, 1 mM ethylenediaminetetraacetic acid (EDTA), 1% sodium dodecyl sulfate, 1% Nonidet P-40, 1:100 proteinase inhibitor cocktail, 50 mM β-glycerophosphate, 50 mM sodium fluoride) (Beyotime, Shanghai, China). The protein concentration was determined with a protein assay kit (Beyotime) following the manufacturer’s instructions. Aliquots of 40 to 50 μg per sample were separated by 10% sodium dodecyl sulfate-polyacrylamide gel electrophoresis (SDS-PAGE), transferred to polyvinylidene fluoride (PVDF) membranes (Millipore, Billerica, MA, USA) and blocked with 5% bovine serum albumin (BSA) in PBST (PBS with 0.1% Tween), then incubated with the following primary antibodies overnight: anti-TLR4, anti-β-actin (Abcam, Cambridge, UK), anti-NFκBp65, anti-phospho-NFκBp65, anti-IκBα and anti-phospho-IκBα (Cell Signaling Technology, Beverly, MA, USA). Then, the membranes were incubated with horseradish peroxidase-conjugated secondary antibody (Boster, Wuhan, China). The blots were visualized using an enhanced chemiluminescence kit (Amersham Biosciences, Piscataway, NJ, USA) according to the manufacturer’s instructions.

### anti-TLR4 antibody/PDTC treatment

The mechanisms involved in the influence of TLR4 ligation on the osteogenic potential of PDLSCs and BMMSCs, were studied using the TLR4 antagonist anti-TLR4 antibody (0.5 μg/ml) and the NF-κB inhibitor pyrrolidinedithiocarbamate (PDTC, 40 ng/ml, Sigma-Aldrich). Cells were seeded in six-well culture plates at a density of 2 × 10^5^ cells/well and expanded in α-MEM (10% FBS) until reaching 80% confluence; then, the culture medium was changed to osteogenic medium with or without LPS (10 μg/ml). For the anti-TLR4 antibody group, anti-TLR4 antibody was added four hours before changing the medium from basal medium to osteogenic induction medium containing LPS (10 μg/ml). For the PDTC group, PDTC was added at the time of changing to the osteogenic medium (containing LPS). Medium was changed every three days. Cells were cultured for 3, 14 and 28 days, and then subjected to assays for western blot or *in vitro* osteogenic differentiation, respectively.

### Induction of experimental periodontitis

Experimental periodontitis was inducted as previously described [19]. All animal procedures were performed according to the guidelines of the Animal Care Committee of the Fourth Military Medical University, Xi’an, China. Twelve adult male Sprague–Dawley rats (SD rats, 250.7 ± 20.5 g, obtained from the Laboratory Animal Research Centre of the Fourth Military Medical University) were used in this protocol. The test group rats were injected with 10 μl of *Escherichia coli* LPS (1 mg/ml) into the maxillary palatal gingiva between the first and second upper molars. The LPS injections were repeated every other day on three separate days. The control group rats received 10 μl of saline injection according to the same schedule as the LPS-injected rats.

### Administration of drug and assessment of alveolar bone loss

Twelve rats were randomly distributed into four groups of three rats each: 1) saline: gingiva was injected with saline; 2) LPS: gingiva was injected with LPS; 3) LPS + anti-TLR4: gingiva was injected with LPS and TLR4 neutralizing antibody (5 μg/ml); and 4) LPS + PDTC: gingiva was injected with LPS and the NF-κB blocking reagent PDTC (400 ng/ml). On day 7, all rats were anesthetized and euthanized by exsanguination. The whole head was removed and the maxillary jaws were scanned and analyzed using a micro-CT system (Siemens Inveon Micro CT, Munich, Germany). The alveolar bone height was measured at 17 different sites (three sites for each of five roots and one site for each root furcation of two teeth) in the maxillary molar regions by recording the distance from the cemento-enamel junction (CEJ) to the alveolar bone crest, using the built-in software. Then, samples were harvested, fixed in 4% paraformaldehyde and decalcified with 5% EDTA before paraffin embedding for further use.

### Immunohistochemistry

To quantify the osteogenic potential of periodontal ligament tissue, the expression of alkaline phosphatase (ALP) was assessed by immunohistochemistry. Paraffin-embedded tissue sections were de-waxed in xylene and rehydrated through graded alcohols to water. Endogenous peroxidase was blocked using 3% H_2_O_2_ for 15 minutes. For antigen retrieval, 0.3% trypsin (Sigma–Aldrich) was used for 15 minutes. Sections were blocked with 10% serum for 30 minutes. Slides were incubated with primary antibody anti-ALP (Abcam, 1:200 dilution) for two hours. Goat anti-rabbit secondary antibody was applied for one hour at room temperature. Sections were then incubated in strept avidin-biotin complex (SABC) (Boster) for 30 minutes. Diaminobenzidine (DAB) solution was applied for two to five minutes and development of the color reaction was monitored microscopically. Slides were counterstained with hematoxylin, dehydrated, cleared and then mounted. The slides were observed under a light microscope (BX-51, Olympus, Japan), and images were acquired using a CDD camera. Quantification of ALP-positive staining was carried out using the software of Image-Pro Plus 6.0.

### Tartrate-resistant acid phosphatase staining of rat alveolar bone section

To quantify osteoclast activity, mature osteoclasts were determined by tartrate-resistant acid phosphatase (TRAP)-positive cells on the bone surface. Deparaffinized sections were refixed with a mixture of 50% ethanol and 50% acetone for 10 minutes. TRAP staining solutions (1.6% naphthol AS-BI phosphate in N, N-dimethylformamide, 0.14% fast red-violet LB diazonium salt, 0.097% tartaric acid, and 0.04% MgCl_2_ in 0.2 M sodium acetate buffer at pH 5.0) were freshly made. The sections were incubated in the solution for 30 minutes at 37°C under a shield and counterstained with toluidine blue. All regents for TRAP staining were purchased from Wako Pure Chemical Industries (Code No. 294–67001, Chuo-ku, Osaka, Japan). The slides were observed under a light microscope (BX-51, Olympus, Tokyo, Japan), and images were acquired using a CDD camera. Quantification of TRAP-positive osteoclasts was carried out by the software of Image-Pro Plus 6.0.

### Statistical analysis

All experiments were repeated at least three times and data are presented as mean ± SD. To test the statistically significant differences between paired observations, the Student’s t-test for paired data was used. All statistical analyses were performed using SPSS software, version 16.0. *P* value <0.05 was considered statistically significant.

## Results

### TLR4 expression of PDLSCs and BMMSCs with or without LPS treatment

**Figure 1:**
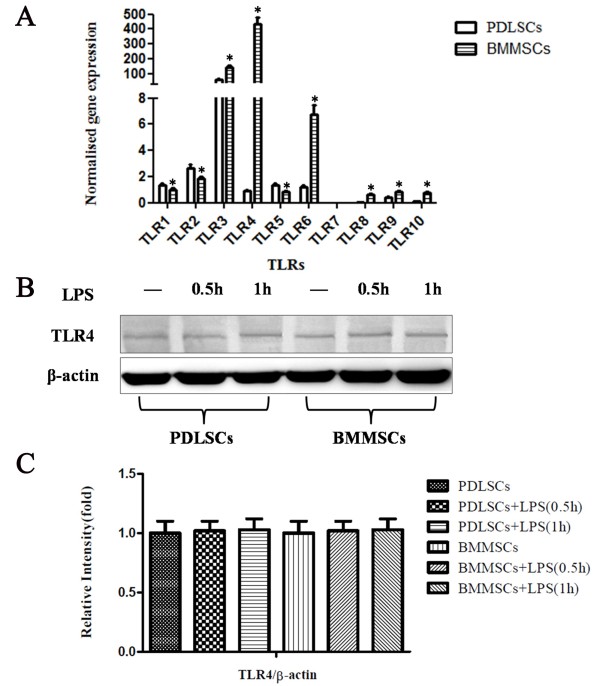
**TLR4 expression of PDLSCs and BMMSCs with or without LPS treatment. (A)** Relative gene expression of TLRs (TLR1 to TLR10) of PDLSCs and BMMSCs was analyzed by qRT-PCR. Relative gene expression of TLRs was determined based on the threshold cycle (CT) values. The expression levels of the target genes were normalized to that of the housekeeping gene β-actin. PDLSCs (*P <0.05 versus PDLSCs, n = 3), BMMSCs (n = 3). **(B)** Western blot analysis showed the protein expression of TLR4, and β-actin was used as the internal control. **(C)** Relative intensity of the tested protein was quantitatively analyzed using the Adobe Photoshop CS2 software. Data represent the means ± SD (n = 3). BMMSCs, bone marrow mesenchymal stem cells; LPS, lipopolysaccharide; PDLSCs, periodontal ligament stem cells; SD, standard deviation; TLR4, Toll-like receptor 4.

To compare the TLRs (TLR1 to TLR10) expression profile of PDLSCs and BMMSCs, we measured their gene expression level by quantitative RT-PCR. Differentially, PDLSCs showed stronger gene expression of TLR1, TLR2 and TLR5 (Figure 1A, *P* <0.05), while BMMSCs showed stronger gene expression for TLR3, TLR4, TLR6, TLR8, TLR9 and TLR10 (Figure 1A, *P* <0.05). In addition, PDLSCs and BMMSCs both showed strong expression of TLR3 and we did not detect the expression of TLR7 in either of them. However, the expression of TLR4 in PDLSCs was much less than that in BMMSCs.To further compare the protein expression of TLR4 in PDLSCs and BMMSCs with or without LPS treatment, Western blot assay was performed to measure the protein expression of TLR4. The results showed that PDLSCs and BMMSCs had similar TLR4 protein expression and TLR4 expression was not changed significantly either at 0.5 or 1 hour after LPS simulation (Figure 1B,C).

### LPS impairs the osteogenic potential of PDLSCs and activates the NF-κB pathway in both PDLSCs and BMMSCs

**Figure 2:**
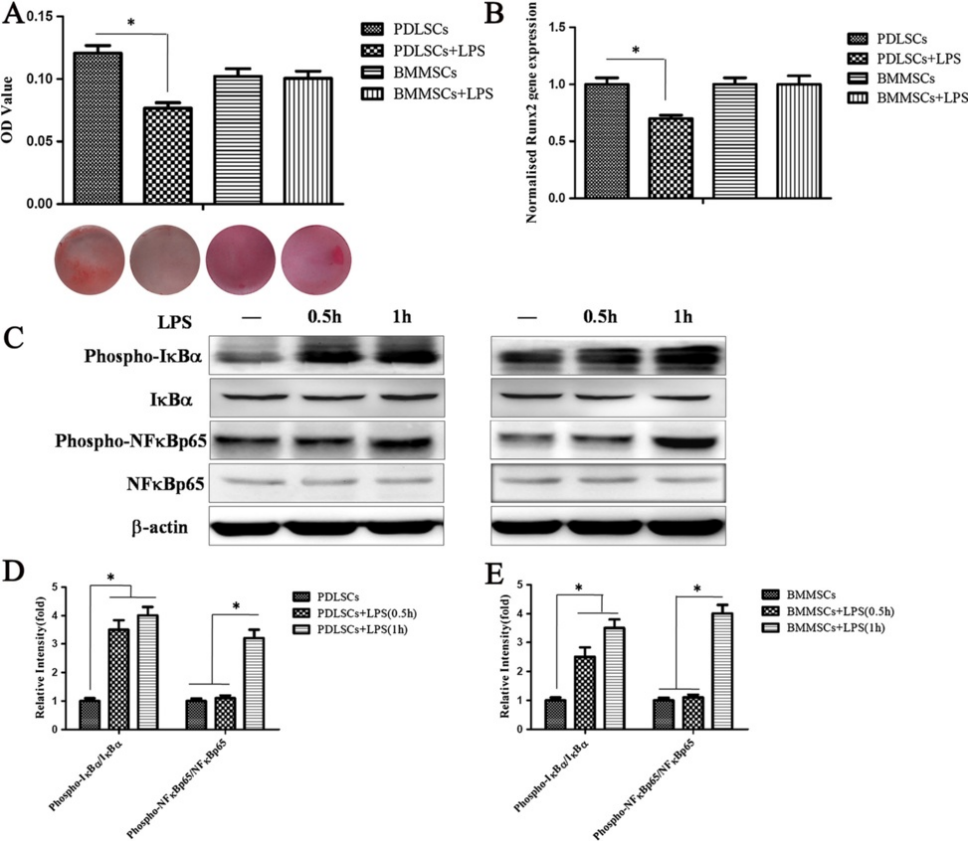
**LPS impairs the osteogenic potential of PDLSCs and activates the NF-κB pathway in PDLSCs and BMMSCs. (A)** PDLSCs and BMMSCs treated with or without LPS were cultured in osteogenic medium and osteogenic differentiation was determined by Alizarin red S staining after 28 days. Alizarin red was then extracted and measured for light absorbance at 620 nm. **(B)** Gene expression of Runx2 in PDLSCs and BMMSCs with or without LPS treatment was measured by qRT-PCR after osteogenic induction for 14 days. **(C)** Western blot analysis showed the protein expression of phospho-IκBα, IκBα, phospho-NFκBp65 and NFκBp65, and β-actin was used as the internal control. **(D, E)** Relative intensity of the tested protein was quantitatively analyzed using Adobe Photoshop CS2 software. Data represent the means ± SD. * *P* <0.05 (n = 3). BMMSCs, bone marrow mesenchymal stem cells; LPS, lipopolysaccharide; NF-κB, nuclear factor κB; PDLSCs, periodontal ligament stem cells; SD, standard deviation.

To identify the role of LPS in regulating the osteogenic differentiation of PDLSCs, LPS (10 μg/ml) was added to the osteogenic induction medium. Alizarin red S staining showed that LPS impaired the osteogenic differentiation ability of PDLSCs, which was demonstrated by the decreased formation of mineralized nodules compared with the control group after four weeks induction (Figure 2A). However, the formation of mineralized nodules of BMMSCs was not changed after LPS treatment, which suggests that LPS did not influence the osteogenic potential of BMMSCs (Figure 2A). LPS also significantly down regulated the expression of the osteogenic related gene *Runx2* in PDLSCs compared with the control group (*P* <0.05, Figure 2B). In accordance with the results of Alizarin red S staining of BMMSCs, the expression of *Runx2* after LPS treatment was not changed significantly compared with the control group (Figure 2B).

The common signaling feature of TLR4 is the activation of the transcription factor nuclear factor-κB (NF-κB). To ascertain the role of LPS in the activation of the TLR4 mediated NF-κB pathway of PDLSCs and BMMSCs, we stimulated PDLSCs and BMMSCs by LPS for 0.5 and one hour, respectively. Western blot analysis showed that the expression of phospho-IκBα in both PDLSCs and BMMSCs was increased after LPS treatment for 0.5 and one hour (*P* <0.05, Figure 2C,D,E). The expression of phospho-NF-κBp65 in both PDLSCs and BMMSCs increased dramatically after LPS treatment for one hour (*P* <0.05, Figure 2C,D,E). Total IκBα and NF-κBp65 were not changed significantly after LPS stimulation (Figure 2C,D,E). Since phosphorylation of IκBα results in the release and nuclear translocation of active NF-κB, these data suggested that LPS can activate TLR4 mediated NF-κB signaling in both PDLSCs and BMMSCs.

### TLR4 or NF-κB blockage reverses the impaired osteogenic differentiation of PDLSCs stimulated by LPS

**Figure 3:**
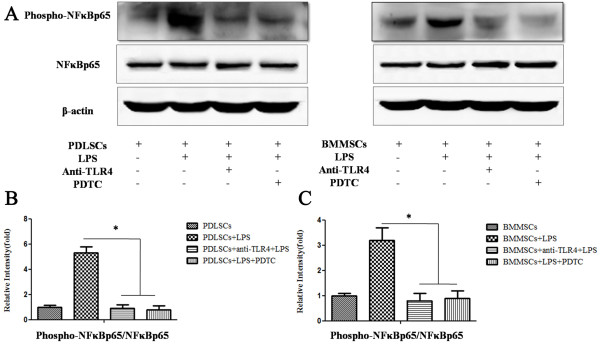
**Anti-TLR4 antibody or PDTC effectively blocks the activated NF-κB pathway activated by LPS. (A)** The TLR4 antagonist anti-TLR4 antibody (0.5 μg/ml) and the NF-κB inhibitor PDTC (40 ng/ml) were incubated with PDLSCs or BMMSCs, which were stimulated by LPS. After 72 hours, western blot showed that the expression of phospho-NFκBp65 was reduced. β-actin was used as the internal control. **(B, C)** The relative intensity of the tested protein was quantitatively analyzed using Adobe Photoshop CS2 software. Data represent the means ± SD. * *P* <0.05 (n = 3). BMMSCs, bone marrow mesenchymal stem cells; LPS, lipopolysaccharide; NF-κB, nuclear factor κB; PDLSCs, periodontal ligament stem cells; PDTC, pyrrolidinedithiocarbamate; SD, standard deviation; TLR4, Toll-like receptor 4.

**Figure 4:**
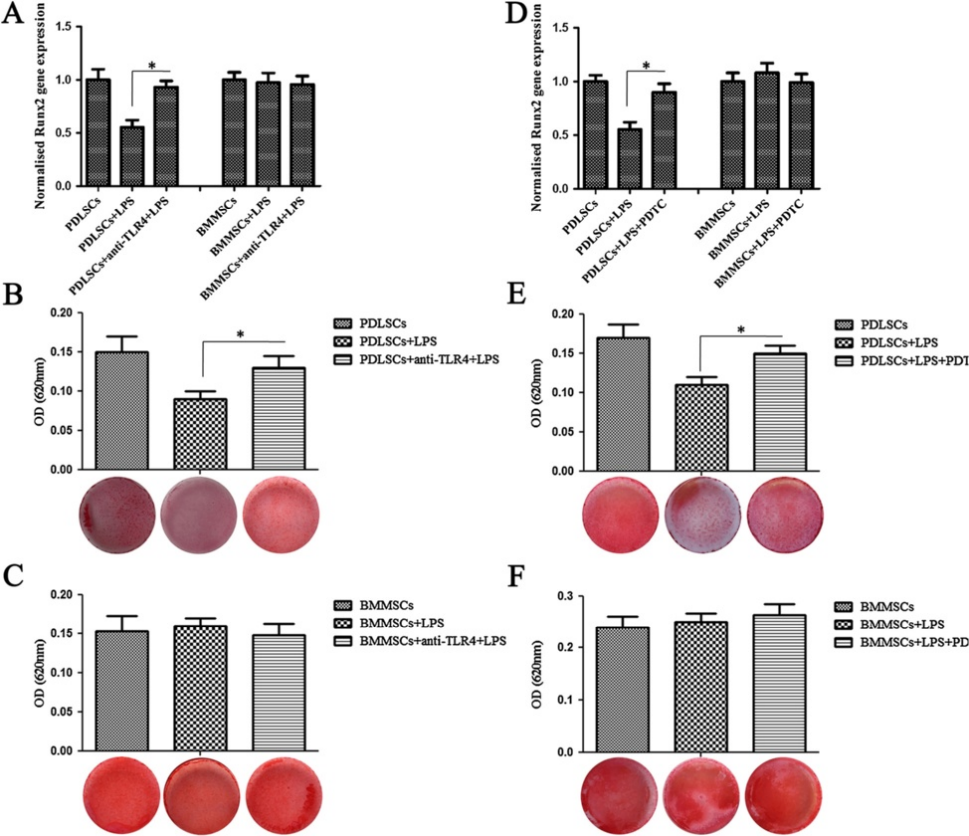
**TLR4 or NF-κB blockage reverses the impaired osteogenic differentiation of PDLSCs stimulated by LPS. (A, D)** Gene expression of Runx2 in PDLSCs and BMMSCs with or without drug treatment was measured by qRT-PCR after osteogenic induction for 14 days. **(B, E)** The impaired osteogenic differentiation of PDLSCs caused by LPS was reversed by anti-TLR4 antibody or PDTC. Osteogenic differentiation was determined by Alizarin red S staining after osteogenic induction for 28 days and alizarin red was then extracted and measured for light absorbance at 620 nm. **(C, F)** The osteogenic differentiation of BMMSCs with or without drug treatment was determined by Alizarin red S staining after osteogenic induction for 28 days. Data represent the means ± SD. *P <0.05 (n = 3). BMMSCs, bone marrow mesenchymal stem cells; LPS, lipopolysaccharide; NF-κB, nuclear factor κB; PDLSCs, periodontal ligament stem cells; PDTC, pyrrolidinedithiocarbamate; SD, standard deviation; TLR4, Toll-like receptor-4.

To document the involvement of TLR4 and its downstream NF-κB signaling pathway in the process of LPS impairment of the osteogenic potential of PDLSCs, anti-TLR4 antibody and the NF-κB inhibitor PDTC were added to the differentiation medium to block the effect of LPS, respectively. Western blot analysis showed that anti-TLR4 antibody or PDTC effectively decreased the expression of phospho-NF-κBp65 in the presence of LPS both in PDLSCs and BMMSCs (*P* <0.05, Figure 3A,B). With respect to the osteogenic differentiation, the gene expression of Runx2 in PDLSCs increased significantly after anti-TLR4 antibody or PDTC treatment (*P* <0.05, Figure 4A,D). Accordingly, the decreased mineralized nodules of PDLSCs following LPS stimulation were also partially reversed after anti-TLR4 antibody or PDTC treatment as assayed by Alizarin red S staining (*P* <0.05, Figure 4B,E).However, the gene expression of Runx2 of BMMSCs was not changed significantly in the presence of anti-TLR4 antibody or PDTC compared with LPS treatment alone (Figure 4A,D), and TLR4 blockage or inhibition of NF-κB did not alter the formation of mineralized nodules of BMMSCs (Figure 4C,F). These results indicated that LPS dampened the osteogenic differentiation of PDLSCs through the TLR4 regulated NF-κB pathway, but not through that of BMMSCs.

### Blockage of TLR4 or NF-κB pathway effectively prevents alveolar bone loss caused by LPS

**Figure 5:**
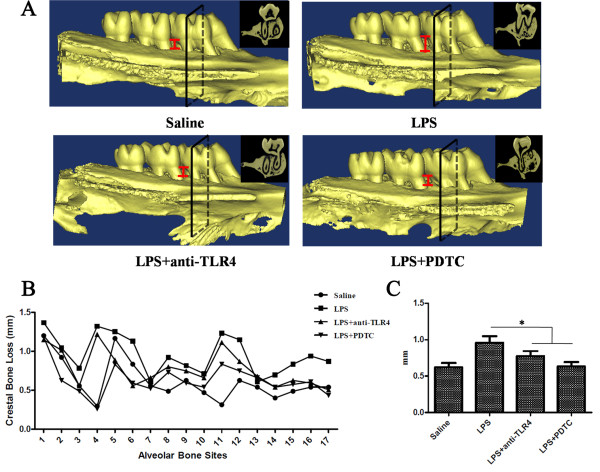
**Blockage of the TLR4 or NF-κB pathway effectively reduces the bone loss caused by LPS *in vivo*. (A)** Bone loss in the saline group, LPS group, LPS + anti-TLR4 group and LPS + PDTC group. **(B)** Alveolar bone loss determination of maxillary molars. Seventeen sites per quadrant (three sites for each of five roots and one site for each root furcation of two teeth) were analyzed morphometrically. **(C)** Mean alveolar bone loss. Data represent the means ± SD. *P <0.05 (n = 3). LPS, lipopolysaccharide; NF-κB, nuclear factor- κB; PDTC, pyrrolidinedithiocarbamate; SD, standard deviation; TLR4, Toll-like receptor 4.

Previous studies have documented that experimental periodontitis can be induced by injection of LPS to SD rats and alveolar bone loss was observed in this model [19,20]. Our results above indicated that blocking TLR4 or NF-κB could enhance the osteogenic differentiation of PDLSCs after LPS treatment. We hypothesized that blockage of TLR4 or NF-κB would also prevent alveolar bone loss in LPS induced experimental periodontitis. Micro-CT analysis showed that LPS can cause obvious alveolar bone loss, and the most obvious bone loss position was just between the first and second maxillary molars, where we injected LPS directly (Figure 5A). Because of the existence of drug diffusion, we tested 17 sites of the first and second maxillary molar regions using a previously described method [19]. We found that, in the LPS group, the distance from the CEJ to the alveolar bone crest at nearly all 17 sites tested was the highest (Figure 5B), which meant that the bone loss in this group was the greatest. With the administration of anti-TLR4 antibody or PDTC, the alveolar bone loss between the first and second maxillary molars was obviously reduced (Figure 5A), and the distance from the CEJ to the alveolar bone crest was reduced (Figure 5B). Statistical analysis of the average bone loss for the 17 sites in each group also showed that anti-TLR4 antibody or PDTC significantly reversed the bone loss caused by LPS (Figure 5C). The average bone loss in each group was: 1) saline = 0.62 ± 0.06; 2) LPS = 0.95 ± 0.07; 3) LPS + anti-TLR4 = 0.77 ± 0.05; and 4) LPS + PDTC = 0.63 ± 0.05 (*P* <0.05, Figure 5C).

### ALP expression is elevated and the osteoclast number is not affected after treatment with anti-TLR4 antibody or PDTC

**Figure 6:**
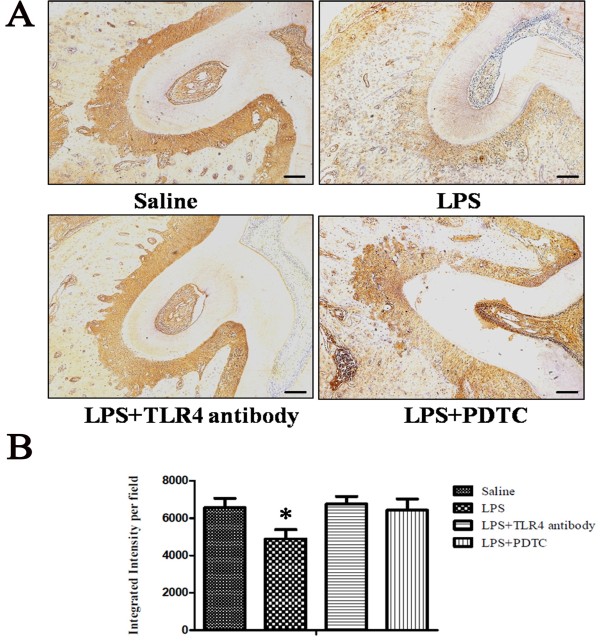
**ALP expression is elevated after treatment with anti-TLR4 antibody or PDTC. (A)** ALP positive staining was seen in the periodontal ligament and its expression in the LPS group was significantly reduced compared to that of the control group. After anti-TLR4 antibody or PDTC administration, the ALP expression was greatly increased and was comparable to that of the control group. **(B)** Quantification of ALP-positive staining. Integrated intensity was measured by Image-Pro Plus 6.0. Scale bar: 125 μm. Data represent the means ± SD. (*P <0.05 versus saline group, n = 3). ALP, alkaline phosphatase; LPS, lipopolysaccharide; PDTC, pyrrolidinedithiocarbamate; SD, standard deviation; TLR4, Toll-like receptor 4.

**Figure 7:**
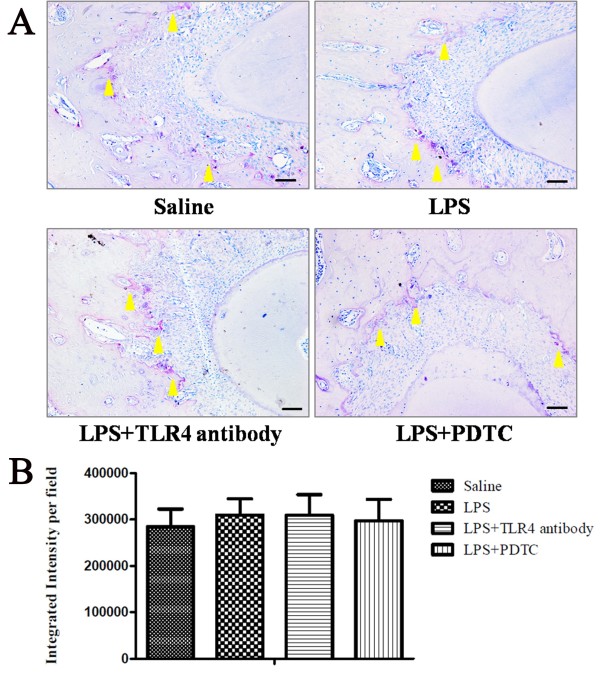
**The number of osteoclasts does not change after anti-TLR4 antibody or PDTC treatment. (A)** TRAP staining showed no significant differences in the number of osteoclasts in the alveolar bone among the saline, LPS, LPS + anti-TLR4 antibody and LPS + PDTC groups. Arrowheads, TRAP-positive osteoclasts (purple cells). **(B)** Quantification of TRAP-positive osteoclasts. Integrated intensity was measured by Image-Pro Plus 6.0. Scale bar: 50 μm. Data represent the means ± SD (n = 3). LPS, lipopolysaccharide; PDTC, pyrrolidinedithiocarbamate; SD, standard deviation; TLR4, Toll-like receptor 4; TRAP, tartrate-resistant acid phosphatase.

To explore if the reduced bone loss was due to the increased osteogenic potential of the periodontal tissue, we used immunohistochemistry staining to detect ALP expression. The result showed positive ALP staining in the periodontal ligament and its expression in the LPS group was significantly reduced compared to that of the control group (*P* <0.05, Figure 6). After anti-TLR4 antibody or PDTC administration, the ALP expression was greatly elevated and was comparable to that of the control group (Figure 6).To further explore if the reduced bone loss was due to the reduced osteoclastogenesis of the periodontal tissue, we checked the number of osteoclasts in the alveolar bone. TRAP staining showed no significant differences in terms of mature osteoclast number among the four groups (Figure 7).

## Discussion

Periodontal regeneration involves the regeneration of cementum, periodontal ligament and alveolar bone. The osteogenic differentiation properties of PDLSCs and BMMSCs are considered to be important during this process. Understanding the factors and mechanisms modulating their capacity for bone regeneration is crucial for the treatment of periodontitis. In this study, we showed that functional TLR4 was expressed by both PDLSCs and BMMSCs. LPS decreased the osteogenic differentiation potential of PDLSCs through a TLR4-mediated NF-κB signaling pathway, but not that of BMMSCs. In addition, blockage of TLR4 or NF-κB signaling prevented the alveolar bone loss caused by LPS, which may provide a new clue for periodontitis therapy.

Host recognition of microbes is largely mediated by TLRs, which are a kind of conserved receptor family including thirteen kinds of TLR subtypes (10 in human and 12 in mice). They recognize a wide variety of pathogen-associated molecular patterns in bacteria, viruses and fungi, as well as certain host-derived molecules [21]. Numerous studies have reported that TLRs are expressed in periodontal tissue and play an important role in immune response and the maintenance of periodontal health [22-25]. Mesenchymal stem cells (MSCs) express TLRs and TLRs ligation can differently affect the functions of MSCs depending on their tissue origin [16,26]. BMMSCs and PDLSCs are two kinds of MSCs from different origins and mediate the regeneration of periodontal tissue. The expression profile of TLRs in PDLSCs and the influence of TLR ligation on these cells have not been studied yet. Thus, in this study, we tested the expression of TLRs on PDLSCs and found that PDLSCs showed different TLR gene expression compared with that of BMMSCs. Our results on the expression profile of TLRs on BMMSCs are in accordance with a previous report [27]. Although PDLSCs showed less TLR4 gene expression compared with that of BMMSCs, we further found that PDLSCs and BMMSCs showed similar protein expression of TLR4. The discrepancy between the gene and protein expression might be caused by different post-transcriptional modification or different degradation speed of the TLR4 protein.

TLR4 recognizes LPS from gram-negative bacteria, which are the most important factor involved in periodontitis. TLR4 ligation can affect the functions of MSCs. However, contradictory results have been reported about the osteogenic differentiation of BMMSCs controlled by LPS/TLR4 signaling. One previous study showed that TLR4 activation had no effect on osteogenic differentiation of human BMMSCs [14]. However, others reported increased osteogenic differentiation of human BMMSCs after LPS stimulation [15]. We found that LPS can activate the TLR4-mediated NF-κB signaling, but the activated NF-κB signaling caused by LPS did not change the osteogenic differentiation of BMMSCs. Our results also showed that the osteogenic differentiation of PDLSCs was impaired by LPS as demonstrated by the decreased formation of mineralized nodules and decreased expression of the osteogenic differentiation gene *Runx2*. This might be caused by different cell origins of these two kinds of MSCs. Furthermore, blockage of TLR4 by TLR4 antibody reversed the impaired osteogenic differentiation of PDLSCs, although not totally. A previous study also showed that TLR4 neutralizing antibody could not produce a 100% blockage of TLR4 even during short-term (four hours) stimulation by LPS [28]. In our study, osteogenic induction was a long *ex vivo* process, which might prevent the antibody fully exerting its effects.

The common signaling feature of TLR4 is the activation of the transcription factor NF-κB, which has been implicated in controlling the expression of inflammatory cytokines and the maturation of inflammatory molecules [29]. We found that LPS activated the NF-κB pathway in both PDLSCs and BMMSCs. Previous research in our lab has indicated that NF-κB signaling plays a central role in regulating the osteogenic differentiation of PDLSCs in inflammatory microenvironments [30]. To ensure the involvement of NF-κB signaling in impairing the osteogenic differentiation of PDLSCs caused by LPS, we used PDTC to block the NF-κB pathway and the osteogenic differentiation of PDLSCs was reversed. However, the osteogenic differentiation of BMMSCs was unchanged in the presence of PDTC. This means that PDTC, even though it can effectively block NF-κB, does not affect the osteogenic differentiation of BMMSCs stimulated by LPS. Interestingly, Hess *et al.*[31] also found that a genetic block of the NF-κB pathway did not interfere with the osteogenic differentiation of BMMSCs. The relationship between the NF-κB pathway and the osteogenic differentiation of MSCs is still controversial [31-35]. In the current study, we found that the NF-κB pathway was activated in both PDLSCs and BMMSCs after LPS treatment, but with different influences on the osteogenic differentiation potential. For allogeneic tissue regeneration, BMMSCs seem like a better candidate for their more stable osteogenic differentiation property as indicated by our results. However, several reports have demonstrated that PDLSCs possess unique periodontal regeneration capacities compared with other MSCs. Fang *et al.*[36] reported that human PDLSCs can generate significant amounts of collagen fibers after being subcutaneously transplanted with a collagen-based gelatin sponge into mice, while BMMSCs did not. Moreover, unlike BMMSCs and dental pulp stem cells, mouse PDLSCs seeded onto tooth root surfaces formed PDL-like tissue, including well-oriented fibers similar to Sharpey fibers [37]. Accordingly, PDLSCs may have an advantage as a promising cell source for functional PDL tissue regeneration, even though their osteogenic potential can be affected by TLR4 ligation.

TLRs act as a double-edged sword and it is still uncertain which specific signaling pathways need to be blocked to attenuate the pathology or enhanced to promote host defense [38]. Here we found that blockage of the TLR4 or NF-κB pathway could partially reverse the osteogenic differentiation of PDLSCs stimulated by LPS *in vitro*. However, the influence of TLR4 or NF-κB blockage *in vivo* on the pathogenesis of periodontitis is still unknown. In this study, we found that the alveolar bone loss caused by LPS was reduced after treatment with TLR4 antibody or NF-κB inhibitor, with elevated ALP expression and unchanged TRAP-staining. Thus, we speculated that the reduced alveolar bone loss was due to the elevated regeneration of the periodontal ligament. Nevertheless, the detailed mechanism still needs further investigation, as the mechanism of LPS induced periodontitis is complicated and not completely understood. It is hypothesized that LPS can activate the host cells in the periodontium, including polymorphonuclear leukocytes, macrophages, fibroblasts and the epithelium, mediated by TLR4 [19]. In addition, LPS can induce alveolar bone loss by stimulating the secretion of proinflammatory cytokines (IL-1β, TNF-α or IL-6) and the formation of osteoclasts [39]. LPS can also induce the secretion of matrix metalloproteinases (MMPs), causing direct damage to periodontal tissues [40]. Although we can not preclude other effects, which reduced the alveolar bone loss, the attempt to block the TLR4 or NF-κB pathway to treat periodontitis shows encouraging results.

No report so far has described the clinical application of the TLR4 antibody, but there is already a phase I study recruiting for assessing the safety of a human anti-TLR4 monoclonal antibody (NI-0101), which is sponsored by NovImmune SA. NF-κB can be activated by TLR4 ligation and because of its pivotal role in inflammation and cell proliferation, much attention has been given to strategies that inhibit NF-κB activity. Most of the drugs that are currently used for treating inflammatory conditions, such as non-steroidal anti-inflammatory drugs (NSAIDs), disease-modifying anti-rheumatic drugs, cyclosporine A and corticosteroids, have inhibitory effects on NF-κB activity [41]. There are also other more highly specific pharmacological NF-κB inhibitors, such as PDTC and flavopiridol [42,43]. Periodontitis is an infectious disease, and how to choose the appropriate TLR4 antibody or NF-κB inhibitors to inhibit the excessive inflammation reaction on the one hand and maintain necessary immune reaction to eliminate pathogens on the other hand is a matter of great concern, which needs further investigation.

## Conclusions

Our research demonstrated that the NF-κB pathway activated by TLR4 ligation mediates the impairment of osteogenic differentiation of PDLSCs. Blockage of TLR4 or the NF-κB pathway can prevent the alveolar bone loss caused by LPS *in vivo*. Our findings suggest that TLR4 or the NF-κB pathway might serve as a new therapeutic target for periodontitis.

## Abbreviations

ALP: alkaline phosphatase; BMMSCs: bone marrow mesenchymal stem cells; BSA: bovine serum albumin; CEJ: cemento-enamel junction; EDTA: ethylenediaminetetraacetic acid; FBS: fetal bovine serum; IL: interleukin; LPS: lipopolysaccharide; MSCs: mesenchymal stem cells; NF-κB: nuclear factor κB; PBS: phosphate-buffered saline; PDL: periodontal ligament; PDLSCs: periodontal ligament stem cells; PDTC: pyrrolidinedithiocarbamate; PVDF: polyvinylidene fluoride; SD rats: Sprague–Dawley rats; TNF: tumor necrosis factor; TLR4: Toll-like receptor 4; TRAP: tartrate-resistant acid phosphatase; α-MEM: α-minimal essential medium.

## Competing interests

The authors declare that they have no competing interests.

## Authors’ contributions

CL, BL and ZD were involved in the practical achievement of these experiments. LG and XH participated in the animal experiment. CL, BL, LL and CH collected, analyzed and interpreted the data. QW and YJ designed the study and provided financial and administrative support. CL and BL wrote the manuscript. QW and YJ revised the manuscript critically for important intellectual content. Each author participated sufficiently in the work to take public responsibility for appropriate portions of the content. All authors read and approved the final manuscript.
